# A Literature Analysis of Consumer Privacy Protection in Augmented Reality Applications in Creative and Cultural Industries: A Text Mining Study

**DOI:** 10.3389/fpsyg.2022.869865

**Published:** 2022-06-29

**Authors:** Yowei Kang, Yu-Sheng Su

**Affiliations:** ^1^Program of Digital Humanities and Creative Industries, National Chung Hsing University, Taichung City, Taiwan; ^2^Department of Computer Science and Engineering, National Taiwan Ocean University, Keelung City, Taiwan

**Keywords:** augmented reality, cultural and creative industry, cross-cultural study, literature review analysis, locational information, museums, privacy, text mining

## Abstract

Digital reality technologies (such as AR, VR, and MR) have recently become a key component of promoting creative and cultural industries (CCIs) worldwide to transform static cultural heritage exhibits into more engaging, entertaining, and immersive experiences. These technologies present an exciting example of studying how consumers would respond to the potential invasion of privacy due to these technologies. This literature review study mainly focuses on one essential branch of CCIs: museums and their applications of digital reality technologies. Because many of these location-based AR applications by museums are inherently sensitive to users’ locational information, there is also a rising concern of the potential infringement of personal privacy (RQ1). A thorough examination of existing literature on how consumers respond to privacy concerns related to the museum’s AR applications will help uncover how scholars have approached and studied these crucial issues in the literature (RQ2). Unlike traditional literature review analyses, we employed a text mining of retrieved 715 studies articles from *Business Source Complete* and *Engineering Village* (E.I.) databases to answer our two research questions. Our study found that privacy and user(s) /visitor(s) has dramatically increased since 2017, echoing the rising concerns of other privacy-invasive technologies. Most notably, key phrases extracted from the literature corpus include “security and privacy,” “privacy and security,” “privacy risks,” “privacy concerns,” “privacy issues,” “user privacy,” “location privacy,” “privacy protection,” and “privacy preserving” that are most pertinent to the rapid implementation of AR technology in the museum sector. Discussions and implications are provided.

## Introduction

Creative and culture industry (CCI) is a term that often refers to industries closely related to the commercialization, creation, distribution, and production of advertisements, cultural heritage, designs, digital games, entertainments, films, musical pieces, museums, and publications (Kang, 2019). Recent definitions of CCIs have included museums as an essential branch of these emerging sectors (Nogare and Murzyn-Kupisz, 2021). Due to the advent of digital reality technologies and during the global COVID-19 pandemic, museums worldwide have taken advantage of these innovations to disseminate museum contents to museum visitors (Kang and Yang, 2022). Some of these digital reality technologies include 360-degree video, augmented reality (AR), mixed reality (MR), virtual reality (VR), and other immersive applications (Cook et al., 2018; Kang, 2019; Lai et al., 2021).

Augmented reality technologies adopted by museums have become a global phenomenon. In comparison with more traditional platforms, digital reality technologies can offer an excellent possibility for collectors, curators, galleries, museums, and the general public to deliver creative and cultural artifacts and interact with them (Javornik, 2016; Farago, 2017; Yang and Kang, 2018; Kang, 2019; Kang and Yang, 2019a,b, 2020). This development has also presented an interesting example to study how consumers would respond to the potential invasion of their privacy (Bruno et al., 2010; Kang, 2019). This literature review study will focus on one essential branch of the creative and cultural industries: museums and their applications of digital reality technologies to help engage visitors with new technology-enabled immersive experiences.

The applications of AR to the museum sector have attracted immense interest from practitioners and researchers. For example, the Providence Academy of the Washing State University in the United States has developed an AR application to engage visitors with the potential to interact with historical figures through their mobile devices (Vogt, 2017; Kang, 2019). Additionally, the American Museum of Natural History allows viewers to interact with life-size dinosaurs models through AR applications (Ang, 2017; Kang, 2019; Kang and Yang, 2020). Easy accessibility of Google Cardboard, Oculus Rift headset, HTC Vive Stream VR, and Samsung Gear VR has helped the promotion of cultural heritage artifacts through these technologies (Ashley, 2017; Kang, 2019; Kang and Yang, 2020; Su et al., 2022). Increasingly, scholars (e.g., Holt, 2018) have proposed the term “virtual trespassing” to address the concern that the superimposed virtual and actual cultural contents have blurred the private space with the public one without prior approval and consent from consumers (Holt, 2018, n.p.; Kang, 2019).

In particular, unlike other digital reality technologies, location-based AR applications by museums must rely on users’ locations to superimpose digitally rendered cultural content with users’ physical locations. As a result, significant concerns about the potential infringement of personal privacy have been brought into the discussion (Aryan and Singh, 2010; Kang and Yang, 2020). These privacy concerns have even been extended to AR facial filters adopted by social media companies (Cowan et al., 2021). This study would argue that privacy perceptions (and the subsequent strategies to protect their privacy) result from how consumers in a particular culture perceive privacy in their unique cultural context. In other words, perceptions of personal privacy and its protection are a cultural phenomenon and will be affected by consumers’ cultural factors. For example, while an individualistic culture like the United States emphasizes personal privacy, a collectivistic culture like Taiwan may place less emphasis on the same privacy concerns (Hofstede, 1991, 2021). According to Triandis (1980, p. 1), cross-cultural psychology focuses on “the systematic study of behavior and experience as it occurs in different cultures, is influenced by culture, or results in changes in existing cultures.” Therefore, it is crucial to examine whether the existing literature on museum’s AR applications has studied how consumers respond to privacy concerns embedded in the location-based AR applications. A thorough investigation of this important topic will help uncover the hidden ecological factors leading to these cross-cultural behavioral differences.

### Objectives of This Study

Because of the great potential of AR technologies among museums worldwide, our study reviewed what has been researched in the existing literature corpus that explores privacy issues related to AR applications by museums to engage museumgoers better. The literature review method has become a popular research approach for digital reality technology researchers in recent years. For example, Guzman et al. (2020) examined the privacy and security impacts of AR and MR technologies, and they claimed their study to be the first study dealing with these critical issues. Similarly, Song et al. (2021) also adopted a literature review method to study the role of AR in digital fabrication. The literature review method is also famous for analyzing existing cross-cultural psychology literature (Obschonka et al., 2022).

Our study employed a literature review approach to situate our study within the cross-cultural psychology perspective and focus on a thorough examination of existing literature to investigate how scholars have studied issues related to how museum consumers deal with these privacy-invasive AR applications. Our approach follows the literature review research conventions to describe and analyze emerging research trends described in our RQ1. However, unlike existing literature in the field that relies on the subjective categorization of research and the articles retrieved from keyword searches (Guzman et al., 2020; Song et al., 2021), we employed a text mining method to extract emerging keywords and key phrases related to the applications of AR technologies by museums from a large number of research articles. Although we have stated our research questions as RQ1 and RQ2, they are related because extracted keywords and key phrases in RQ1 will contribute to our analyses for RQ2.

To summarize, our study aims to answer the following research questions:

Research Question 1 (RQ1): What are the critical privacy concerns related to the augmented reality technology applications of the museum sector, as demonstrated in the text mining analyses of existing literature?

Research Question 2 (RQ2): How would we account for the existing literature’s research trends that deal with consumers’ privacy concerns?

## Literature Review

Privacy has been defined as consumers’ anticipated concerns about the potential loss of their privacy (Xu et al., 2011; Kang and Yang, 2020, 2021a). Xu et al. (2011) have proposed that the concept of privacy is difficult to define; however, it is mainly related to a sense of control (Thompson et al., 2019). Because of the integration of digital reality technologies by the museum sector in recent years, privacy-related topics have grabbed the attention of many researchers and museum practitioners (Kang and Yang, 2020). Among these digital reality applications, AR technologies by museums that rely on visitors’ location privacy pose the most significant challenges to museum visitors’ location privacy. Given the global diffusion of AR applications among museums, the study of cross-cultural privacy concerns among consumers has also been done internationally. For example, according to the 2016 KMPG survey that studies how cross-cultural consumers perceive privacy issues, fifty-five percent of global consumers report that they are most concerned about the misuse of personal information for unsolicited marketing activities, the sale of personal information to 3rd party companies, and the absence of cyber-security mechanisms (KMPG, 2016; Kang and Yang, 2020). KMPG’s survey data also confirmed our speculation that perceptions of privacy, despite their concerns over individual privacy, are a universal phenomenon; country-unique variations may exist. For example, 39% of consumers from China (39%) are the most concerned about their online privacy, followed by approximately the same percentage of consumers from India (35%) and Singapore (32%).

The use of museum visitors’ location information also echoes rising concerns about possible misusing among global digital media users (We Are Social, 2020). In another global survey by We Are Social (2020), 64% of global Internet users (between 16 and 64 years old) reported that they were worried about how companies may use their data. Similar to KMPG’s study, cross-cultural variations also exist. For example, Central and Southern Americans have shown that they are most concerned about these privacy-invasive technologies. Among these countries, the most concerned countries are Columbia (80%), Brazil (79%), and Mexico (79%) (We Are Social, 2020). Due to its collectivistic culture (Hofstede, 1991, 2021), consumers in Eastern Asia countries are less concerned about personal privacy: South Korea (40%) and Japan (40%) (Kang and Yang, 2021b; We Are Social, 2020).

Past research has studied the effects of privacy concerns in different media platforms on consumers’ consumption and usage behaviors (Lips et al., 2017; Kang and Yang, 2021b). For example, Kang and Yang (2021b) have studied the impacts of privacy concerns on social media use. They proposed a convergence-divergence interpretation to speculate whether technologies may homogenize or heterogenize cross-cultural consumer behaviors. Another empirical study by Duan and Dholakia (2015) also has confirmed that social media usage among contemporary Chinese users has transformed Chinese value systems from “suppressing desire, delaying gratification and thriftiness” (p. 409). Additionally, social media usage also helps converge different social strata in Chinese society by merging the values of elite and grass-root Chinese users (Duan and Dholakia, 2015).

Scholarly interests in applying cross-cultural perspectives have been found in studying global health emergencies such as the COVID-19 pandemic (Chin et al., 2022a), international business models (Chin et al., 2021), and responsible innovation development (Chin et al., 2022b). More pertinent to the present study, Nam and Kannan (2020) proposed their conceptual paper on how cross-cultural differences may affect consumer journeys in the digital marketplace. They proposed that motivation, social interaction and brands, emerging technologies, channel choice, and privacy could be affected by the characteristics of cross-cultural consumers. Similarly, Dogruel (2019) studied the perceptions and acceptance of nudges in making people aware of their privacy and found that, compared with the United States, German consumers are more receptive to state nudges as an intervention to increase awareness of personal privacy. Thompson et al. (2019) similarly studied cultural dimensions as proposed by Hofstede (1991, 2021) (such as collectivism/individualism, power distance, masculinity/femininity, long-term orientation, and uncertainty avoidance indices) and their potential impacts on consumers’ privacy concerns, and their trust in government surveillance mechanisms.

Trepte et al. (2017) studied the privacy calculus framework and confirmed that consumers with higher levels of collectivism would place a higher emphasis on privacy protections to safeguard their collective community. Their cross-national survey data of over 1,600 participants (from China, Germany, the Netherland, the United Kingdom, and the United States) has confirmed the importance of cultural factors on cross-cultural variations of privacy perceptions. For example, the uncertainty avoidance index (Hofstede, 1991; Kang and Yang, 2021b) turned out to be the most crucial predictor affecting consumers’ risk and benefit calculation in their decision to control their personal information (Trepte et al., 2017; Kang and Yang, 2021b). Additionally, consumers’ individualism (Hofstede, 1991) was negatively associated with their willingness to avoid risks; consumers from a high individualistic culture tend to welcome risks (Trepte et al., 2017; Kang and Yang, 2021b).

Zabihzadeh et al. (2019) empirically confirmed the relationships between consumer culture and their perceptions of privacy. They studied 200 participants from Iran and the United States and found that their perceptions of privacy are closely related to individualism and collectivism conceptualized by Hofstede (1991). Additionally, for Iranian consumers, family, home, *Aberoo* (i.e., saving face), and safety rank the top four associations with privacy, while for United States consumers, safety, security, personal, and secret are the top four words.

Unfortunately, a careful examination of the existing museum and AR literature has confirmed a gap in a systematic examination of AR applications by many museums that have adopted mobile location-sensitive AR technologies to exhibit their cultural contents via visitors’ smartphones (Kang and Yang, 2020). As a result, the present discussions on privacy-preserving or privacy design enable consumers to control and decide their privacy. Concepts (such as decisional privacy) have emerged (Kang and Yang, 2021a).

## Research Method

### Combining Text Mining and Literature Review Methods

The literature review method has been popular in digital reality technology research (Guzman et al., 2020) and has been increasingly adopted by cross-cultural psychology researchers (Obschonka et al., 2022). While these early literature review studies focus on the categorization of the technologies and their related accessories, such as head-mounted devices, the “value-sensitive approach” has emerged in 2000 (Friedman and Kahn, 2003) to investigate data ownership, integrity, privacy, and secrecy issues (refer to Guzman et al., 2020, p. 110:3–110:4 for a complete review). After an extensive keyword search of existing popular databases, our study is the first study to use a text mining study to explore how the existing literature is studying potential privacy infringement as one of the most noticeable social impacts of AR technologies by museums (Franziska et al., 2014; Kang and Yang, 2020).

As a recently emerged method for scholars to study a variety of phenomena with a minimum amount of human interference (Lin et al., 2016; Yang and Kang, 2018; Kang and Yang, 2022), this objective data categorization and interpretation approach can address problems that we have found in traditional literature review analysis and meta-analysis studies (Guzman et al., 2020; Song et al., 2021). Additionally, a text mining method also offers researchers the excellent opportunity to systematically review and analyze many retrieved articles objectively, without human errors (Lin, cited in Yang and Kang, 2018). For example, Song et al. (2021) analyzed only 84 articles. Our study, on the contrary, studied 715 articles from keyword searches. In the cross-cultural psychology field, Obschonka et al. (2022) studied merely 79 articles collected in 2020. Therefore, we argue that the extent and the scope of our literature review method are comparable to existing studies.

### The Compilation of Literature Corpus and Sample Characteristics

This text-mining literature analysis study used *QDA Miner* and *WORDStat* to analyze emerging keywords and key phrases in the literature corpus. QDA Miner can easily analyze a large number of documents “in a variety of file formats including M.S. Word, WordPerfect, RTF, PDF, HTML, XML, MS Access, Excel, SPSS, Paradox, dBase, QSR N6, Nvivo, Atlas.ti, HyperResearch, Ethnograph, Transana, Transcriber” (LaPan, 2013: 775).

We have chosen these two databases to compile our literature corpus because of their comprehensive coverage of business- and engineering-related topics. *Business Source Complete* covers research areas in accounting, advertising, banking, management, and marketing, while *Engineering Village (E.I.)* includes over 190 subject areas in engineering research. We used the keyword phrases [“augmented reality” and “privacy” and “museums”] to search these two databases. The results have generated zero articles in both databases, suggesting the innovativeness of our research topic. To refine our searches, we revised our key phrases to [“augmented reality” and “privacy”] (1st keyphrase pair) and [“augmented reality” and “museums”] (2nd keyphrase pair). We generated 44 articles from *Business Source Complete* from the first pair and 21 from the 2nd pair. Using *Engineering Village (E.I.)* database, we generated 237 articles from the 1st pair, while 413 articles were retrieved from the 2nd pair. We compiled a literature review corpus of 715 articles from 1996 to 2022, covering peer-reviewed journal publications, conference papers, conference proceedings, etc. We used the abstracts of these articles because they summarize the essence of each article.

Based on the text mining technique, we analyzed recurrent keywords and critical phrases, following the procedures by Miner (2012) and Kang and Yang (2022). Additionally, the algorithms in the QDA Miner include K-Means Clustering for text categorization and text-to-numeric transformation and Latent Dirichlet Allocation (LDA) for topic modeling (Valcheva, n.d.) that are most relevant to this study. For example, we first conducted the document-clustering algorithm to transform unstructured textual data in each retrieved abstract into a structured representation before applying this procedure to reduce the vector space through either direct clustering or dimensionality reduction (Miner, 2012).

## Results and Discussion

### Results From the WordCloud Analyses

We conducted several text mining techniques to provide empirical data to answer our first question. Our second research question aims to identify recurrent keywords and key phrases from the retrieved literature review corpus that studies the AR applications in the museum sector to demonstrate the research trends in the related topics. These techniques include the extraction of keywords and key phrases to estimate and demonstrate their relative importance among a-bag-of-words model in a Natural Language Processing algorithm in the corpus by examining Term-Frequency (T.F.) or TF-IDF (Term-Frequency-Inverse document Frequency) that shows the frequency of these terms (Teso et al., 2018; Yang and Kang, 2018; Kang and Yang, 2022). T.F. or TF-IDF statistics help researchers identify reoccurring words or phrases viewed as more important and prominent (Kang and Yang, 2022). A visualization of the word frequency statistics is a Word cloud analysis technique that has been a widely used text mining technique to demonstrate the frequency of keywords and key phrases in a graphical manner (Srivastava, 2014; Kang and Yang, 2022). As seen in Table 1 below, several keywords related to AR applications by museums have emerged; these include “privacy” (TF-IDF = 226.1), “security” (TF-IDF = 169.2), “data” (TF-IDF = 194.1), “information” (TF-IDF = 184.9), and location (TF-IDF = 157.0) (Refer to Table 1). Also noteworthy is the reoccurrence of consumer-related keywords, such as “user” (TF-IDF = 201.9), “users” (TF-IDF = 191.7), “visitors” (TF-IDF = 186.7), “experience” (TF-IDF = 171.5), “experiences” (TF-IDF = 112.8), and “visitor” (TF-IDF = 102.0) (Refer to Table 1).

**TABLE 1:** Extracted key words from the literature corpus.^1, 2, 3^

	Frequency	% Total	No. Cases	% Cases	TF-IDF
System	412	0.36%	197	27.55%	230.7
Privacy*	477	0.42%	240	33.57%	226.1
Learning	284	0.25%	128	17.90%	212.2
Applications	377	0.33%	198	27.69%	210.2
Computing	189	0.16%	56	7.83%	209.1
Mobile	387	0.34%	211	29.51%	205.1
User*	439	0.38%	248	34.69%	201.9
Data*	324	0.28%	180	25.17%	194.1
Users*	352	0.31%	204	28.53%	191.7
Cultural	288	0.25%	155	21.68%	191.2
Information*	404	0.35%	243	33.99%	189.4
Visitors*	318	0.28%	185	25.87%	186.7
Technology	358	0.31%	229	32.03%	177.0
Technologies	320	0.28%	201	28.11%	176.4
Experience*	314	0.27%	198	27.69%	175.1
Security*	195	0.17%	97	13.57%	169.2
Heritage	218	0.19%	122	17.06%	167.4
Application	283	0.25%	191	26.71%	162.2
Digital	245	0.21%	156	21.82%	162.0
Smart	166	0.14%	76	10.63%	161.6
Devices	233	0.20%	149	20.84%	158.7
Design	245	0.21%	163	22.80%	157.3
Location*	166	0.14%	81	11.33%	157.0
Content	200	0.17%	124	17.34%	152.2
Systems	189	0.16%	119	16.64%	147.2
Objects	177	0.15%	106	14.83%	146.7
Exhibition	154	0.13%	84	11.75%	143.2
Model	160	0.14%	94	13.15%	141.0
Interactive	192	0.17%	136	19.02%	138.4
Interaction	188	0.16%	136	19.02%	135.5
Edge	101	0.09%	33	4.62%	134.9
Development	173	0.15%	127	17.76%	129.8
Display	132	0.12%	76	10.63%	128.5
Game	116	0.10%	58	8.11%	126.5
Cloud	97	0.08%	37	5.17%	124.8
Environment	163	0.14%	124	17.34%	124.0
Device	127	0.11%	79	11.05%	121.5
Time	153	0.13%	116	16.22%	120.8
Physical	142	0.12%	103	14.41%	119.5
App	106	0.09%	56	7.83%	117.2
Provide	160	0.14%	134	18.74%	116.4
Proposed	130	0.11%	92	12.87%	115.8
Present	159	0.14%	136	19.02%	114.6
Experiences*	130	0.11%	97	13.57%	112.8
Work	144	0.13%	118	16.50%	112.7
Public	112	0.10%	71	9.93%	112.3
Visual	111	0.10%	75	10.49%	108.7
Guide	96	0.08%	53	7.41%	108.5
People	121	0.11%	91	12.73%	108.3
Access	105	0.09%	67	9.37%	108.0
Social	112	0.10%	78	10.91%	107.8
Services	102	0.09%	63	8.81%	107.6
Approach	123	0.11%	97	13.57%	106.7
Project	106	0.09%	74	10.35%	104.4
Video	93	0.08%	54	7.55%	104.3
Context	126	0.11%	109	15.24%	102.9
Visitor*	100	0.09%	68	9.51%	102.2
Method	98	0.09%	66	9.23%	101.4
Participants	94	0.08%	60	8.39%	101.2
Navigation	74	0.06%	31	4.34%	100.9
Network	85	0.07%	47	6.57%	100.5

^1^Keywords such as “virtual,” “AR,” “augmented reality,” “museums,” and “museum” has been removed. ^2^Only keywords with a TF-IDF value larger than 100 will be kept in the table. ^3^Keywords relevant to privacy issues related to museum visitors are remarked with *.

### Results From Key Phrase Exaction

The key phrase extraction procedure in *QDA Miner* and *WordStat* is to extract the most salient phrases in the literature corpus useful for “document categorization, clustering, indexing, search, and summarization” (Kang and Yang, 2020). As seen in Table 2 below, the key phrases related to the study of consumers’ concerns over privacy-related issues about AR applications by museums, “security and privacy” (TF-IDF = 169.5) is among the most prominent phrases in the compiled corpus, followed by “privacy concerns” (TF-IDF = 61.7), “privacy protection” (TF-IDF = 48.2), “location privacy” (TF-IDF = 46.8), “privacy and security” (TF-IDF = 25.2), “privacy risks” (TF-IDF = 24.4), “user privacy” (TF-IDF = 21.8), and “privacy preserving” (TF-IDF = 22.1) (Refer to Table 2). Our study’s significant findings demonstrate the scope, extent, and topology of consumers’ privacy concerns regarding museums’ AR applications in the literature. For example, while it is anticipated that “privacy concerns” emerge as a repetitive key phrase, this study also observes that more specific privacy concerns have emerged, ranging from “location privacy,” “user privacy,” and “privacy and security.” Additionally, the phrase “privacy preserving” has emerged to demonstrate how engineers investigate the “privacy by design” principle to ensure consumer privacy protection.

**TABLE 2:** Extracted key phrases from the literature corpus.^1, 2^

	Frequency	No. Cases	% Cases	TF- IDF
Cultural heritage	147	92	12.87%	130.9
Mobile devices	61	47	6.57%	72.1
Security and privacy*	55	39	5.45%	69.5
Cloud computing	49	14	1.96%	83.7
Internet of things	49	40	5.59%	61.4
Privacy concerns*	44	29	4.06%	61.2
Head mounted	33	32	4.48%	44.5
User experience*	33	28	3.92%	46.4
Edge computing	32	15	2.10%	53.7
Privacy protection*	31	20	2.80%	48.2
Mobile application	29	24	3.36%	42.7
Smart glasses	28	15	2.10%	47.0
User interface	27	20	2.80%	41.9
Artificial intelligence	24	22	3.08%	36.3
Fog computing	24	7	0.98%	48.2
Location privacy*	24	8	1.12%	46.8
Mobile device	23	19	2.66%	36.2
Internet of things IoT	22	22	3.08%	33.3
Access control	20	11	1.54%	36.3
Mobile applications	20	17	2.38%	32.5
Location based services*	19	11	1.54%	34.4
Privacy issues*	19	17	2.38%	30.9
Google glass	18	17	2.38%	29.2
Proposed system	18	15	2.10%	30.2
Social media	18	18	2.52%	28.8
Deep learning	17	8	1.12%	33.2
Digital technologies	17	16	2.24%	28.1
Informal learning	17	12	1.68%	30.2
Low latency	17	17	2.38%	27.6
Mobile app	17	11	1.54%	30.8
Low cost	16	14	1.96%	27.3
Guidance system	15	9	1.26%	28.5
Large scale	15	14	1.96%	25.6
Machine learning	15	11	1.54%	27.2
Physical objects	15	12	1.68%	26.6
Privacy and security*	15	15	2.10%	25.2
Visitors experience*	15	13	1.82%	26.1
Wide range	15	14	1.96%	25.6
Article presents	14	14	1.96%	23.9
Computation offloading	14	2	0.28%	35.7
Guide system	14	10	1.40%	26.0
Privacy risks*	14	13	1.82%	24.4
Visitor experience*	14	13	1.82%	24.4
Archaeological sites	13	10	1.40%	24.1
Computer vision	13	12	1.68%	23.1
Design process	13	12	1.68%	23.1
Eye tracking	13	7	0.98%	26.1
Natural history	13	10	1.40%	24.1
Digital information	12	11	1.54%	21.8
Head mounted displays	12	12	1.68%	21.3
Navigation system	12	8	1.12%	23.4
QR codes	12	9	1.26%	22.8
Smart toys	12	4	0.56%	27.0
User privacy*	12	11	1.54%	21.8
Audio guides	11	11	1.54%	19.9
Big data*	11	10	1.40%	20.4
Case studies	11	11	1.54%	19.9
Cutting edge	11	8	1.12%	21.5
Decision making	11	5	0.70%	23.7
Design and implementation	11	11	1.54%	19.9
Exhibition system	11	6	0.84%	22.8
Indoor positioning	11	8	1.12%	21.5
Information technology	11	11	1.54%	19.9
Learning experience	11	10	1.40%	20.4
Microsoft hololens	11	10	1.40%	20.4
Personal information*	11	6	0.84%	22.8
Privacy preserving*	11	7	0.98%	22.1
Sensor data	11	7	0.98%	22.1
Context aware	10	9	1.26%	19.0
Continuous sensing	10	4	0.56%	22.5
Digital content	10	8	1.12%	19.5
Display system	10	9	1.26%	19.0
Energy consumption	10	5	0.70%	21.6
Future work	10	10	1.40%	18.5
High level	10	8	1.12%	19.5
Historical relics	10	2	0.28%	25.5
Human computer interaction	10	10	1.40%	18.5
Immersive experiences*	10	6	0.84%	20.8
Lessons learned	10	10	1.40%	18.5
Location information*	10	9	1.26%	19.0
Point of view	10	9	1.26%	19.0
Points of interest	10	8	1.12%	19.5
Sensitive information*	10	8	1.12%	19.5
Smart devices	10	9	1.26%	19.0
User interaction*	10	9	1.26%	19.0
Visual information	10	7	0.98%	20.1

^1^Only keyphrase with a minimum frequency of 10 will be kept in the table. ^2^Keyphrases relevant to privacy issues related to museum visitors are remarked with *.

Another significant finding from our text mining research is to investigate further the privacy concerns related to the utilization of consumers’ location privacy information in offering AR museum-going experiences by interacting with AR-superimposed exhibitions or on-location museum guidance. Our study has also discovered another remarkable group of key phrases in the corpus about the location information related to AR applications in the museum sector. For example, “location-based services” (TF-IDF = 34.2), “personal information” (TF-IDF = 22.8), “location information” (TF-IDF = 19.0), and “sensitive information” (TF-IDF = 19.5). Additionally, the emphasis on how museum users or visitors would respond to these concerns has emerged as a major research topic. The terms “location-based services,” “location information,” and “personal information” have emerged. Additionally, it is interesting to note the relationship between location information and “sensitive information.”

Furthermore, despite the privacy concerns of these AR applications in the museum sector, the literature corpus has demonstrated that museum curators and professionals attempt to make the best use of AR technologies to generate more positive museum experiences. Reoccurring key phrases include “user experience” (TF-IDF = 46.4), “visitors experience” (TF-IDF = 26.1), “visitor experience” (TF-IDF = 24.4), “immersive experiences” (TF-IDF = 20.8), and “user interaction” (TF-IDF = 19.0) (Refer to Table 2). The TF-IDF statistics quantify the importance of a key phrase in the overall document corpus (Silge and Robinson, 2019, 2022). The statistics demonstrate privacy-related issues consumers perceive in the museum sector studied in the existing literature.

### Research Trend Analysis

**FIGURE 1:**
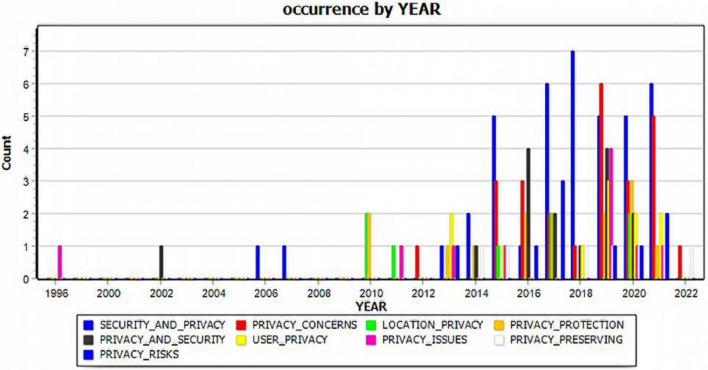
Trend analysis of privacy-related publications between 1996 and 2022.

Trend analysis has been used along with conventional literature review to assess the journal metrics (Kokol, 2017) and to compare methodologies used in academic studies over a long period and across geographical regions (Umer et al., 2018). Unlike the human coding of collected 153 service marketing articles (Umer et al., 2018), our study demonstrates that a text mining literature review can objectively analyze many articles (*N* = 715). While Umer et al. (2018) focused on methodology, geographic regions, and years of publications, one of the significant findings from our study is that we have examined research themes and variations between 1996 and 2022 and have visually presented that the emergence of key research topics from our literature corpus. For example, extracted key phrases that are most pertinent to our study include “security and privacy,” “privacy and security,” “privacy risks,” “privacy concerns,” “privacy issues,” “user privacy,” “location privacy,” “privacy protection,” and “privacy preserving” (Refer to Figure 1 below). The visualization of the numerical data contributes to our understanding that privacy-related issues have emerged significantly since 2010, with their highest growth after 2016.

**FIGURE 2:**
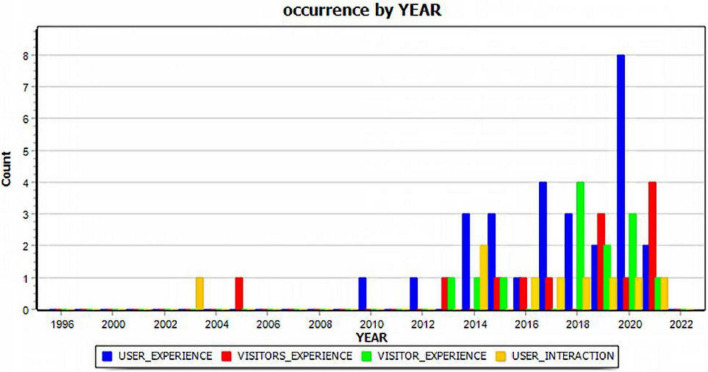
Trend analysis of museum users/visitors-related publications between 1996 and 2022.

The following year-by-year analyses of selected key phrases present another significant finding of our study to allow researchers to comprehend the research trends as demonstrated in the existing literature. It is noteworthy that, while research on “privacy issues” first appeared in 1996, followed by sporadic research on “privacy and security” (2002), “security and privacy” (2006, 2007), the exponential growth of the privacy-related literature can be found after 2015 when the number of related articles jumped to 12. Privacy issues related to AR applications have since been gaining interest among scholars, with the total publications reaching 11 in 2016, 17 in 2027, 11 in 2018, 25 in 2019, 20 in 2020, and 18 in 2021. The rapid increase of these publications demonstrates AR technologies on consumer privacy. Extracted key phrases related to how consumers would respond to the implementation of AR technologies by museums and its impacts on users’ experiences include “user experience,” “user interaction,” “visitors experience,” and “visitor experience” (Refer to Figure 2 below). While research on “visitor experience” first appeared in 2005, scholarly interest in user-related topics increased in 2014 with 6 articles, followed by 5 in 2015, 6 in 2017, 8 in 2019, 13 in 2020, and 8 in 2021. In particular, research on visitor experience has increased from one article in 2015, 2 in 2013, 4 in 2018, 5 in 2019, 4 in 2020, and 5 in 2021. The increase of these publications demonstrates that AR technologies by museums have been related to their impacts on visitor experiences. The demonstration of research topic variations offers museum scholars and practitioners a road map to develop their research and application strategies when integrating these innovative AR technologies.

## Conclusion

### Summary

As demonstrated in this text mining study of privacy literature related to the implementations of AR in the museum sector, our literature review study has confirmed the growing importance of these topics in the past 5 years. Our study summarizes what research topics have been gaining attention among scholars. The increase in publications after 2015 has collided with the rapid deployment of AR technologies. With the heightened security and privacy concerns among consumers, new research topics such as “privacy preserving” in system design emerged in 2014, along with more research on “privacy protection.” Additionally, the growing focus on visitor(s) experience has echoed the implementation of AR technologies to enhance museum visitors’ experiences inside the museums. However, the unintended and unanticipated negative impacts have infringed on consumers’ privacy. This text mining study of existing literature on these essential topics has offered scholars a longitudinal analysis of trends in research topics. Unfortunately, digressing from our original plan to locate and analyze cross-cultural psychological research on consumers’ perceptions and strategies to address location privacy invasion due to AR applications in museums, our study shows an ample opportunity for psychological scholars to study this global phenomenon.

### Limitations and Future Research Directions

Like any literature review study, several limitations need to be considered to interpret better and understand our research findings. First, the thoroughness of sampled articles in our literature review corpus is a primary concern in any text mining research (Yang and Kang, 2018; Kang and Yang, 2019a, 2021b, 2022). This study only retrieved articles from the widely used social scientific *Business Source Complete* and technology-oriented *Engineering Village* (E.I.) databases. Despite our intention to be comprehensive, future scholars may generate more comprehensive findings by examining other databases. Second, another limitation is processing words, keywords, key phrases, and lexicon dictionaries to extract recurrent linguistic patterns and trends through a bag-of-words approach (Teso et al., 2018; Kang and Yang, 2021b). Some text-mining scholars have claimed that the heavy dependence on a single word may fail to address the diversity of word meanings (i.e., polysemy) (Teso et al., 2018; Kang and Yang, 2021b).

Additionally, the lack of contextual information related to each keyword and key phrase may not probably address the relative importance of each research topic (Teso et al., 2018; Yang and Kang, 2018; Kang, 2019; Kang and Yang, 2021b). Scholars may explore the “keyword in context” function in QDA Miner can benefit their research. Lastly, this study only collected and analyzed studies published in the English language and archived in the selected commercial databases. Studies published outside of these linguistic and database parameters were not text mined. Future scholars from other language regions may benefit from analyzing non-English academic research to shed light on these issues.

## Data Availability Statement

The original contributions presented in this study are included in the article/supplementary material, further inquiries can be directed to the corresponding author.

## Author Contributions

Both authors listed have made a substantial, direct, and intellectual contribution to the work, and approved it for publication.

## Conflict of Interest

The authors declare that the research was conducted in the absence of any commercial or financial relationships that could be construed as a potential conflict of interest.

## Publisher’s Note

All claims expressed in this article are solely those of the authors and do not necessarily represent those of their affiliated organizations, or those of the publisher, the editors and the reviewers. Any product that may be evaluated in this article, or claim that may be made by its manufacturer, is not guaranteed or endorsed by the publisher.

## References

[B1] Ang B. (2017). *Augmented Reality In The Classroom: Move Over, Pokemon Go, It’s Time For Science Class; Augmented Reality, The Technology Used In The Mobile Game, Is Now Being Used In Education And Design, Among Other Things. The Strait Times.* Available online at: https://www.straitstimes.com/lifestyle/augmented-reality-in-the-classroom-move-over-pokemon-go-its-time-for-science-class [Accessed July 21, 2019].

[B2] Aryan A. Singh S. (2010). “Securing location privacy in augmented reality,” in *Proceedings of the 2010 5th International Conference on Industrial and Information Systems*, Mangalore. 10.1109/ICIINFS.2010.5578714

[B3] Ashley L. (2017). *Facing The Future: How Museums Are Embracing New Trends And Technologies. Lifestyle.* Available online at: https://www.thenational.ae/lifestyle/family/facing-the-future-how-museums-are-embracing-new-trends-and-technologies-1.82108 [Accessed September 8, 2019].

[B4] Bruno F. Bruno S. De Sensi G. Luchi M.-L. Mancuso S. Muzzupappa M. (2010). From 3D reconstruction to virtual reality: a complete methodology for digital archaeological exhibition. *J. Cult. Herit.* 11 42–49. 10.1016/j.culher.2009.02.006

[B5] Chin T. Meng J. Wang S. Y. Shi Y. Zhang J. X. (2022a). Cross-cultural metacognition as a prior for humanitarian knowledge: when cultures collide in global health emergencies. *J. Knowl. Manage.* 26 88–100. 10.1108/JKM-10-2020-0787

[B6] Chin T. Caputo F. Shi Y. Calabrese M. Aouina-Mejri C. Papa A. (2022b). Depicting the role of cross-cultural legitimacy for responsible innovation in Asian-Pacific business models: a dialectical systems view of Yin-Yang Harmony. *Corp. Soc. Responsib. Environ. Manage.* 1–11. 10.1002/csr.2303

[B7] Chin T. Shi Y. Rowley C. Meng J. W. (2021). Confucian business model canvas in the Asia Pacific: a Yin-Yang harmony cognition to value creation and innovation. *Asia Pacific Bus. Rev.* 3 1–17. 10.1080/13602381.2020.1795481

[B8] Cook A. V. Jones R. Raghavan A. Saif I. (2018). *Digital Reality: The Focus Shifts From Technology To Opportunity: Tech Trends 2018. Deloitte Insights.* Available online at: https://www2.deloitte.com/us/en/insights/focus/tech-trends/2018/immersive-technologies-digital-reality.html [Accessed September 9, 2019].

[B9] Cowan K. Javornik A. Jiang P. (2021). Privacy concerns when using augmented reality face filters? explaining why and when use avoidance occurs. *Psychol. Market.* 38, 1799–1813. 10.1002/mar.21576

[B10] Dogruel L. (2019). Privacy nudges as policy interventions: comparing the U.S. and German media users’ evaluation of information privacy nudges. *Inf. Commun. Soc.* 22 1080–1095. 10.1080/1369118X.2017.1403642

[B11] Duan J. Y. Dholakia N. (2015). The reshaping of Chinese consumer values in the social media era: exploring the impact of Weibo. *Qual. Market Res.* 18 409–426. 10.1108/QMR-07-2014-0058

[B12] Farago J. (2017). *Virtual Reality Has Arrived In The Art World. Now What? The New York Times.* Available online at: https://www.nytimes.com/2017/02/03/arts/design/virtual-reality-has-arrived-in-the-art-world-now-what.html [Accessed July 20, 2019].

[B13] Franziska R. Kohno T. O. Molnar D. (2014). Security and privacy for augmented reality systems. *Commun. ACM* 57 88–96. 10.1145/2580723.2580730 29973196

[B14] Friedman B. Kahn P. H. Jr. (2003). “Human values, ethics, and design,” in *The Human-Computer Interaction Handbook: Fundamentals, Evolving Technologies, and Emerging Applications*, ed. Sears J. J. A. (Mahwah, NJ: Lawrence Erlbaum Associates), 1177–1201.

[B15] Guzman J. A. Thilakarathna K. Seneviratne A. (2020). Security and privacy approaches in mixed reality: a literature survey. *ACM Comput. Surveys* 52 110–137. 10.1145/3359626

[B16] Hofstede G. H. (1991). *Cultures And Organizations: Software Of The Mind.* New York, NY: McGraw-Hill.

[B17] Hofstede G. H. (2021). *Country Comparison. Hofstede Insights.* Available online at: https://www.hofstede-insights.com/country-comparison/taiwan,the-usa/ [Accessed February 27, 2021].

[B18] Holt S. (2018). *How Augmented Reality is Changing The Museum Experience.* Available online at: https://www.psfk.com/2018/04/museum-ar-art.html [Accessed February 27, 2021].

[B19] Javornik A. (2016). Augmented reality: research agenda for studying the impact of its media characteristics on consumer behaviour. *J. Retailing Consum. Serv.* 30 252–261. 10.1016/j.jretconser.2016.02.004

[B20] Kang Y. W. (2019). “The applications of digital reality in creative and oceanic cultural industries: the case of Taiwan,” in *Cases on Immersive Virtual Reality Techniques*, ed. Yang K. C. C. (Hershey, PA: IGI-Global Publisher), 269–296. 10.4018/978-1-5225-5912-2.ch012

[B21] Kang Y. W. Yang K. C. C. (2019a). “What do facebook users feel about facebook advertising? Using an experiential sampling method (ESM) to study their digital advertising experiences,” in *Impacts of Online Advertising on Business Performance*, eds Semerádová T. Weinlich P. (Hershey, PA: IGI-Global Publisher), 1–27. 10.4018/978-1-7998-1618-8.ch001

[B22] Kang Y. W. Yang K. C. C. (2019b). “Employing digital reality technologies in art exhibitions and museums: a global survey of best practices and implications,” in *Virtual and Augmented Reality in Education, Art, and Museum*, eds Guazzaroni G. Pillai A. S. (Hershey, PA: IGI-Global Publisher), 139–161. 10.4018/978-1-7998-1796-3.ch008

[B23] Kang Y. W. Yang K. C. C. (2020). “Privacy concerns in the V.R. and A.R. applications in creative cultural industries: a text mining study,” in *Managerial Challenges and Social Impacts of Virtual and Augmented Reality*, ed. Loureiro S. M. C. (Hershey, PA: IGI-Global Publisher), 142–164. 10.4018/978-1-7998-2874-7.ch009

[B24] Kang Y. W. Yang K. C. C. (2021a). “Exploring mobile users’ daily experiences in the United States and Taiwan: an experience sampling method to study privacy concerns in location-based mobile marketing applications,” in *Privacy and Security Challenges in Location Aware Computing*, eds Saravanan P. S. Balasundaram S. R. (Hershey, PA: IGI-Global Publisher), 1–25. 10.4018/978-1-7998-7756-1.ch001

[B25] Kang Y. W. Yang K. C. C. (2021b). “Will social media and its consumption converge or diverge global consumer culture?,” in *Analyzing Global Social Media Consumption*, ed. Wamuyu P. K. (Hershey, PA: IGI-Global Publisher), 68–87. 10.4018/978-1-7998-4718-2.ch005

[B26] Kang Y. W. Yang K. C. C. (2022). “Framing digital reality technology application by museums during covid-19 pandemic: a comparative text mining research,” in *Extended Reality Usage During COVID-19 Pandemic*, eds Pillai A. S. Guazzaroni G. (Cham: Springer-Verlag), 109–125. 10.1007/978-3-030-91394-6_8

[B27] KMPG (2016). *Companies That Fail To See Privacy As A Business Priority Risk Crossing The ‘Creepy Line’. KMPG.* Available online at: https://home.kpmg/sg/en/home/media/press-releases/2016/11/companies-that-fail-to-see-privacy-as-a-business-priority-risk-crossing-the-creepy-line.html [Accessed June 23, 2019].

[B28] Kokol P. (2017). Trend analysis of journal metrics: a new academic library service? *Res. Commun.* 105 240–242. 10.5195/jmla.2017.98 28670211 PMC5490701

[B29] Lai Y. H. Chen S. Y. Lai C. F. Chang Y. C. Su Y. S. (2021). Study on enhancing AIoT computational thinking skills by plot image-based VR. *Interact. Learn. Environ.* 29 482–495. 10.1080/10494820.2019.1580750

[B30] LaPan C. (2013). Review of QDA miner. *Soc. Sci. Comput. Rev.* 31 774–778. 10.1177/0894439313492711

[B31] Lin F.-R. Hao D. Liao D. C. (2016). Automatic content analysis of media framing by text mining techniques. *Paper presented at the 2016 49th Hawaii International Conference on System Sciences*, Koloa, HI. 10.1109/HICSS.2016.348

[B32] Lips A. Miriam B. Eppel E. A. (2017). Understanding and explaining online personal information-sharing behaviours of New Zealanders: a new taxonomy. *Inf. Commun. Soc.* 20 428–443. 10.1080/1369118X.2016.1184697

[B33] Miner G. (2012). *Practical Text Mining and Statistical Analysis for Non-structured Text Data Applications.* Cambridge, MA: Academic Press.

[B34] Nam H. Y. Kannan P. K. (2020). Digital environment in global markets: cross-cultural implications for evolving customer journeys. *J. Int. Mark.* 28 28–47. 10.1177/1069031X19898767

[B35] Nogare C. D. Murzyn-Kupisz M. (2021). Do museums foster innovation through engagement with the cultural and creative industries? *J. Cult. Econ.* 45 671–704. 10.1007/s10824-021-09418-3

[B36] Obschonka M. Cai Q. Y. Athena C. Y. Chan S. M. Sydni A. J. B. Lee S. K. (2022). International psychological research addressing the early phase of the COVID-19 Pandemic: a rapid scoping review and implications for global psychology. *Int. J. Psychol.* 57 1–19. 10.1002/ijop.12823 34904220

[B37] Silge J. Robinson D. (2019). *Text Mining With R: A Tidy Approach.* Sebastopol, CA: O’Reilly.

[B38] Silge J. Robinson D. (2022). *Term Frequency and Inverse Document Frequency (TF-IDF) Using Tidy Data Principles. Tidytext.* Available online at: https://juliasilge.github.io/tidytext/articles/tf_idf.html (accessed February 27, 2021).

[B39] Song Y. Koeck R. Luo S. (2021). Review and analysis of augmented reality (a.r.) literature for digital fabrication in architecture. *Autom. Constr.* 128:103762. 10.1016/j.autcon.2021.103762

[B40] Srivastava T. (2014). *Build A Word Cloud Using Text Mining Tools Of R. Analytics Vidhya.* Available online at: https://www.analyticsvidhya.com/blog/2014/05/build-word-cloud-text-mining-tools/ [Accessed April 25, 2019].

[B41] Su Y. S. Cheng H. W. Lai C. F. (2022). Study of virtual reality immersive technology enhanced mathematics geometry learning. *Front. Psychol.* 13:760418. 10.3389/fpsyg.2022.760418 35250708 PMC8892099

[B42] Teso E. Olmedillab M. Martínez-Torresc M. R. Toral S. L. (2018). Application of text mining techniques to the analysis of discourse in eWOM communications from a gender perspective. *Technol. Forecast. Soc. Change* 129 131–142. 10.1016/j.techfore.2017.12.018

[B43] Thompson N. McGill T. Bunn A. Alexander R. (2019). Cultural factors and the role of privacy concerns in acceptance of government surveillance. *J. Assoc. Inf. Sci. Technol.* 71 1129–1142. 10.1002/asi.24372

[B44] Trepte S. Reinecke L. Ellison N. B. Quiring O. Yao M. Z. Ziegele M. (2017). A Cross-cultural perspective on the privacy calculus. *Soc. Media Soc.* 3 1–13. 10.1177/2056305116688035 33090108

[B45] Triandis H. C. (1980). Reflections on trends in cross-cultural research. *J. Cross Cult. Psychol.* 11 35–58. 10.1177/0022022180111003

[B46] Umer M. Razi S. Wright L. T. (2018). Analyzing research methodologies and publication trends in service marketing literature. *Cogent Bus. Manage.* 5:1446265. 10.1080/23311975.2018.1446265

[B47] Valcheva S. (n.d.). *Text Mining Algorithms List. IntellSpot.* Available online at: https://www.intellspot.com/text-mining-algorithms/ (accessed February 27, 2021).

[B48] Vogt T. (2017). *The Name Is Now, Simply: The Historic Trust Group Declares New Name, New Focus.* Vancouver, WA: The Columbian, A1.

[B49] We Are Social (2020). *Digital 2020.* Available online at: https://wearesocial.com/us/blog/2020/01/digital-2020-us/ [Accessed March 21, 2020].

[B50] Xu H. Dinev T. Smith J. (2011). Information privacy concerns: linking individual perceptions eith institutional privacy assurances. *J. Assoc. Inf. Syst.* 12 798–824. 10.17705/1jais.00281

[B51] Yang K. C. C. Kang Y. W. (2018). “Integrating virtual reality and augmented reality into advertising campaigns: history, technology, and future trends,” in *Encyclopedia of Computer Graphics and Games*, eds Lee N. Wu X.-M. El Rhalibi A. (New York, NY: Springer), 1–9. 10.1007/978-3-319-08234-9_132-1

[B52] Yang K. C. C. Kang Y. W. (2019). “Augmented, mixed, and virtual reality applications in cause-related marketing (CRM),” in *Cases on Immersive Virtual Reality Techniques*, ed. Yang K. C. C. (Hershey, PA: IGI-Global Publisher), 217–240. 10.4018/978-1-5225-5912-2.ch010

[B53] Zabihzadeh A. Ali Mazaheri M. Hatami J. Nikfarjam M. R. Panaghi L. Davoodi T. (2019). Cultural differences in conceptual representation of “privacy”: a comparison between Iran and the United States. *J. Soc. Psychol.* 159 357–370. 10.1080/00224545.2018.1493676 30095370

